# Periampullary tumors in a patient with pancreatic divisum and neurofibromatosis type 1: a case report

**DOI:** 10.1186/s13053-023-00262-4

**Published:** 2023-09-29

**Authors:** Bin-bin Li, Hui Zheng, Yi-Dan Lou, Wen-Wei Zhang, Song Zheng

**Affiliations:** 1 The Fourth School of Clinical Medicine, Zhejiang Chinese Medical University, Hangzhou, 310053 Zhejiang China; 2 Department of Medical Oncology, Affiliated Hangzhou First People’s Hospital, Zhejiang University School of Medicine, Hangzhou, 310000 Zhejiang China; 3 Zhejiang University School of Medicine, Hangzhou, China; 4 Department of Medical Oncology, Affiliated Hangzhou Cancer Hospital, Zhejiang University School of Medicine, Hangzhou, 310000 Zhejiang China; 5 Key Laboratory of Clinical Cancer Pharmacology and Toxicology Research of Zhejiang Province, Hangzhou, 310000 Zhejiang China

**Keywords:** NF1, Neuroendocrine tumors, GIST, Pancreas divisum, Periampullary adenoma, ERCP

## Abstract

**Introduction:**

We present a case of a male patient with neurofibromatosis type 1 diagnosed with pancreatic divisum and several gastrointestinal tumors. A 55-year-old man was admitted to the hospital with recurrent chronic pancreatitis, indicating a large mass in the ampulla. In addition, genetic testing revealed two unique germline mutations in the neurofibromin *(NF1)* gene, and their potential interaction in promoting cancer was further investigated.

**Conclusion:**

The first similar case was reported in 2020. The current case was distinct from other cases since an additional two *NF1* mutations were found in the patient. In conjunction with prior case reports, our findings imply that genetic testing in patients diagnosed with neurofibromatosis type 1 could be helpful in the development of effective treatments.

## Introduction

Neurofibromatosis type 1 (NF1) is a hereditary condition inherited in an autosomal dominant manner [1, 2]. An NF1 diagnosis requires the fulfillment of two out of seven criteria. These are the presence of six or more café-au-lait macules (CALMs) of at least 5 mm in size before adolescence and more than 15 mm after adolescence, two or more neurofibromas of any type, or one plexiform neurofibroma, freckling in the axillary or inguinal regions, optic pathway glioma(OPG), two or more iris Lisch nodules, unique bone lesions (e.g., sphenoid wing dysplasia, long bone cortical thinning with or without pseudoarthrosis), and first-degree relatives who meet the NF1 standard [3]. As axillary freckling, CALMs, and neurofibromas are frequent skin symptoms that may lead to a diagnosis, evaluating the effectiveness of intervention and prenatal counseling, even within the same family, can be challenging due to variances in expression patterns [4].

A literature review revealed that neurocutaneous syndrome NF1 is characterized by benign and malignant neoplasms [4]. While cutaneous neurofibromas typically remain benign [5], they are often complicated by gastrointestinal tumors several years after the diagnosis of NF1 [6]. The appearance of gastrointestinal stromal tumors (GISTs) and neuroendocrine tumors (NETs) surrounding the ampulla is regarded as an incredibly indicative and even pathological characteristic of NF1 [1]. Only one case of NF1 with periampullary adenocarcinoma and pancreas divisum (PD) has been reported earlier [7]. We present a case involving various malignant tumors (NF1, GIST, periampullary adenoma, NETs, and PD) and intend to investigate their patient correlations in the present study.

## Case report

**Fig. 1:**
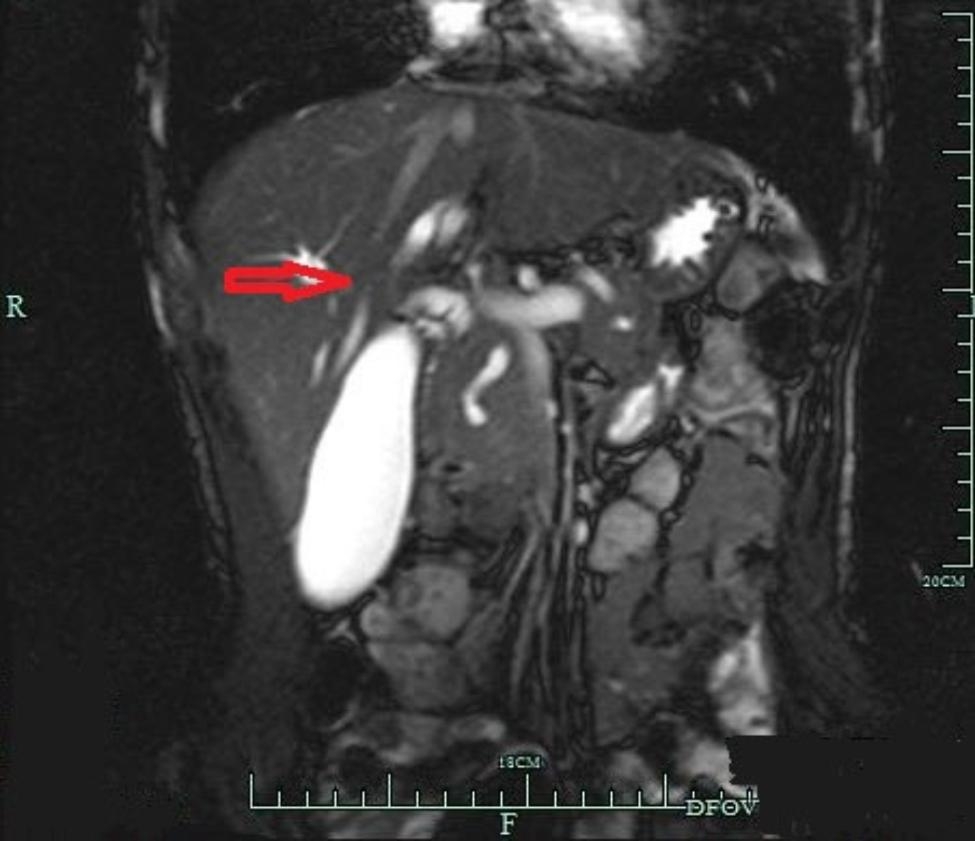
Magnetic resonance cholangiopancreatography: diagnosis of pancreas divisum

**Fig. 2:**
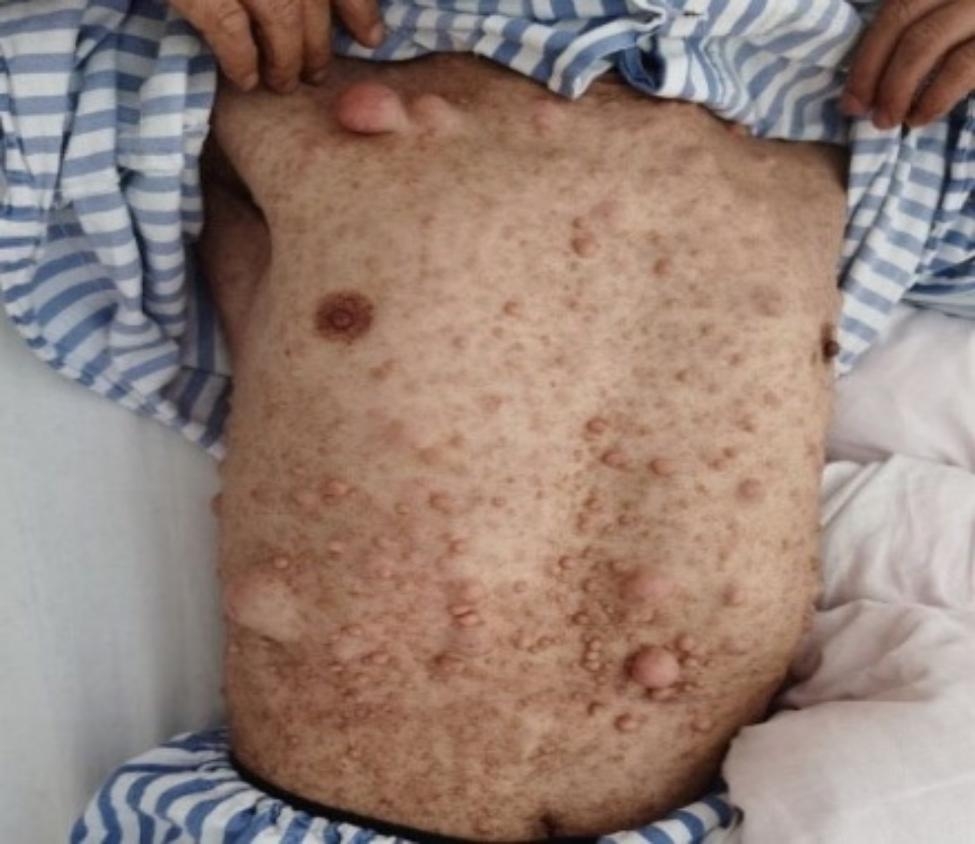
The symptoms of NF1: multiple flat, light-brown patches of skin pigment, skin-fold freckling, and visible neurofibromas under the skin

A 55-year-old man was admitted to the hospital on March 1, 2021, with the primary complaint being abdominal pain that lasted for more than a year. Due to persistently elevated blood amylase levels, the patient had undergone endoscopic retrograde cholangiopancreatography (ERCP) five years earlier, and PD was discovered during the procedure **(**Fig. 1**)**. Body examinations were performed in the hospital **(**Fig. 2**)**, detecting neurofibromas, CALMs, and skin-fold freckling over the whole body, which were diagnosed as PD and NF1. Further examinations showed a blood amylase level of 1592 U/l (normal values, 25–125 U/l), while abdominal computed tomography (CT) scans revealed pancreatic duct dilatation, and re-ERCP indicated a duodenal ampulla mass. Multiple subcutaneous nodules were observed on the chest, neck, abdomen, and back. Magnetic resonance imaging (MRI) of the head showed no abnormalities.

There were indications for surgical excision on July 7, 2020, when a pathological biopsy detected fragmented mucosal tissue in the duodenum with moderate-severe epithelial dysplasia. On January 19, 2021, proximal jejunectomy, pylorus-preserving pancreaticoduodenectomy (Whipple) with routine reconstruction, and cholecystectomy were conducted with the patient’s and his family’s consensus. Macroscopic examination of the Whipple specimen revealed a 3.5 cm*1.5 cm*1 cm tumor in the ampulla that had invaded the subserosal connective tissue and pancreatic tissues, and metastasis was found in the peripancreatic lymph nodes (4/12) (including 1 adenocarcinoma metastasis, 2 NETs, and 1 adenocarcinoma + NET). During the procedure, a third multinodular tumor with a diameter of 0.3 cm was discovered in the small intestine wall; the specimen submitted indicated low-grade GIST.

**Fig. 3:**
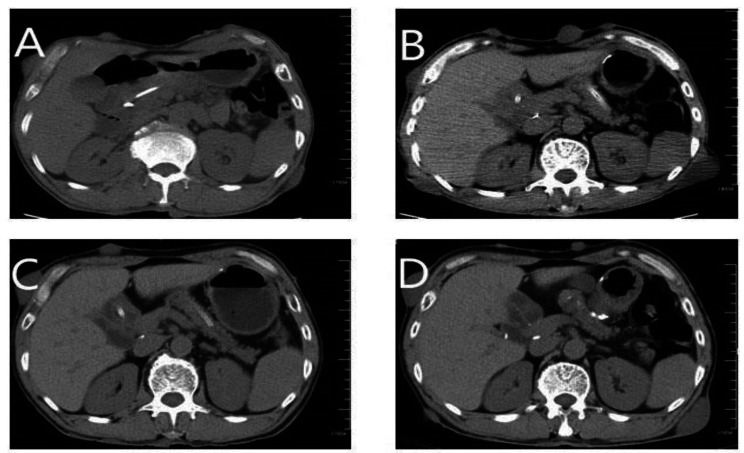
Upper abdomen puncture scan. **(A)** Before surgery: the ampulla of Vater was indistinct, the pancreas was plump, and the contours were clear. (**B)** Two months after Whipple surgery: bleeding and edema in the surgical area. **(C)** Reexamination after 8 cycles of CAPEOX chemotherapy: no abnormal enhancement focus at the gastrointestinal anastomosis after cholecystectomy. **(D)** Reexamination on 2023.02.02: no abnormal enhancement focus in the

The patient’s recovery from surgery went properly. Following the recommendations of the NCCN, the patient underwent 8 cycles of oxaliplatin plus capecitabine (CAPEOX chemotherapy regimen) due to numerous lymph node metastases (PS = 0). Capecitabine monotherapy was started as maintenance therapy on August 20, 2021, and continues to date **(**Fig. 3**)**. Relevant indicators were regularly reexamined, and no tumor progression or new tumor was found.

The resected tumor tissues were subjected to genetic testing. This identified a germline frameshift mutation in the neurofibromin (*NF1*) gene (NM_001042492: exon10: p. I377fs) with an allele frequency of 47.79% and an *NF1* germline termination mutation (NM_001042492: Exon23: p. L1018X) with an allele frequency of 11.18%. Neither of these *NF1* mutations has been reported in any population database or publication. It was impossible to conduct genetic testing on the patient’s family history because the patient’s parents had passed away, and the patient had never been married and had no siblings.

## Discussion

NF1 is the most common neurocutaneous syndrome, with a frequency of 1 in 2500 in the overall population [4]. Neurofibromatosis may increase the risk of multiple tumors [8]. The development of benign and malignant tumors is characterized by NF1, with the largest chance of developing symptomatic visceral tumors occurring within the first six years of life [9]. CALMs and overall skin pigmentation are the earliest signs, appearing within the first two years of life [10]. CALMs in the skin folds resemble freckles and are referred to as Crowe’s sign, the most specific indication for NF1 [11]. Excision may not be possible for people who have an excessive number of cutaneous neurofibromas. According to a Finnish study, at the age of 50, NF1 has a cumulative cancer risk of 38% and a lifetime cancer risk of 59.6%. However, equivalent values for the general population are 3.9% and 30.8%, respectively [12]. Because of this, a proper diagnosis is crucial at the prefrontal stage of a tumor and offers useful prediction guidance for tumor management [4].

This case report describes a patient who was identified with various neoplasms (NETs, GIST, and ampullary adenocarcinoma). The GIST and NETs were perhaps related to NF1, and the diagnosis was verified through germline genetic testing. Among the tumors identified, GIST is the most frequently observed gastrointestinal tumor in patients with NF1, accounting for 5–25% of all NF1 tumors [6, 13]. However, NF1-related GISTs show significant differences from sporadic GISTs. First, sporadic GISTs are mainly located in the stomach (50–70%), while NF1-related GISTs are mostly found in the small bowel (68%), and most NF1-related GISTs are of the spindle-cell type (75%), for which the prognosis is better than that of sporadic GISTs [14]. Second, mutations in the KIT and PDGFRA genes are rarely documented in NF1-associated GISTs, which are less malignant and risky tumors, and thus, imatinib treatment is usually unnecessary [13]. In addition to diagnosing NF1, the present patient was identified with an anatomical anomaly known as PD. PD is a congenital hypoplasia characterized by the absence or inadequate fusion of the ventral (main or Wirsung) and dorsal (marginal or Santorini) ducts during pancreatic development. It is most commonly observed on MRI or MRCP in patients with chronic pancreatitis [15, 16]. With a 10% frequency in the general population, PD is the most prevalent congenital abnormality of the pancreas. In approximately 5% of patients, it can manifest symptoms. Terumi et al. [17] collected 32 cases of PD, with 12.5% of the cases observed to have pancreatic tumors. Histological examination of the related pancreatic cancer revealed adenocarcinoma, with all tumors developing in the dorsal pancreas. Takuma et al. [18] examined 104 cases of PD and found that all pancreatic tumors originated from the dorsal pancreas and showed varying degrees of dorsal pancreatic duct dilatation. They hypothesized that long-term pancreatic duct obstruction induced by relative narrowing of the minor papilla could be a factor in oncogenesis. Although some NF1 individuals with periampullary tumors have been found to have PD, most of these people do not have any symptoms of the disease. According to Bertin et al. [19], the frequency of PD does not differ significantly between patients with idiopathic pancreatitis and those with no pancreatic disease (5%vs.7%), demonstrating that PD alone is not a cause of pancreatitis. According to a recent study, the risk for people with PD, idiopathic pancreatitis, and mutations in the CFTR gene is 12 times higher than that for people without those mutations. This simple PD should not be regarded as the pathogenesis of acute pancreatitis (AP)/chronic pancreatitis (CP) [20, 21]. The association between CFTR mutations or polymorphisms and PD might explain why the occurrence of both PD and CFTR mutations may develop into pancreatitis. However, no evidence exists that this developmental abnormality is directly related to cancer [15, 19].

The NF-1 gene is located at chromosome 17q11.2 [7, 22]. Neurofibromin, its gene product, is a tumor suppressor that inhibits RAS-GTP in the Ras-mediated signaling pathway. Neurofibromin inhibits cell proliferation pathways such as MAPK/ERK kinase (MEK), mitogen-activated protein kinase (MAPK), and cyclic adenosine monophosphate (cAMP)-mediated protein kinase A (PKA) [23, 24]. Patients with NF1 do not have a properly functional neurofibromin protein, which results in unregulated activation of RAS-GTP pathways, which leads to aberrant proliferation and the development of cancer [10, 25]. Although this autosomal dominant disorder is fully penetrant in adulthood, variable expressivity is common within families. This can be attributed to the wide range of mutations found in the *NF1* gene [4]. The NF1 gene, which spans 350 kb of the genome, is highly vulnerable to many forms of mutations. Approximately 85–90% of these mutations are point mutations, 5–10% are microdeletions, and 2% are exon deletions or duplications [26]. Approximately 80% of the described mutations result in premature termination codons and truncation of neurofibromin [26–28]. The present patient carried a termination mutation in exon 23 of the *NF1* gene c.3052delT as well as a frameshift mutation in exon 10, c.1131del1134del.pI377fs, which predicts the occurrence of a premature stop codon and abnormal gene arrangement, resulting in the loss of all functional domains of the neurofibromin protein, which may be the cause of gastrointestinal tumors. Furthermore, Perrone hypothesized that in NF1 patients, increased susceptibility of both epithelial tissue (endocrine or exocrine) and nonepithelial tissue around the ampulla (endocrine or exocrine) leads to a higher risk of tumorigenesis and that extensive tumor excision is frequently required in treatment [29].

## Conclusion

We identified two novel germline frameshift mutations in the *NF1* gene that contribute to the relationship among NF1, GIST, NETs, and ampullary adenocarcinoma. In this case, factors associated with PD and pathogenic mutations in *NF1* may have acted synergistically to increase the risk of multiple tumors. A gastrointestinal tumor should be considered if individuals with NF1 mutations suffer gastrointestinal symptoms. It is also recommended that genetics in these patients assist in the development of effective treatment regimens.

## Data Availability

The raw data supporting the conclusions of this article will be made available by the authors without undue reservation.

## Abbreviations

NF1: Neurofibromatosis type 1
CALMs: café-au-lait macules
OPG: optic pathway glioma
NETs: Neuroendocrine tumors
GIST: Gastrointestinal Stromal Tumor
PD: pancreas divisum
ERCP: Endoscopic retrograde cholangiopancreatography
CT: Computed tomography
MRI: Magnetic Resonance Imaging
MRCP: Magnetic resonance cholangiopancreatography
Whipple: proximal jejunectomy, pylorus-preserving pancreaticoduodenectomy
AP: acute pancreatitis
CP: chronic pancreatitis
